# A Multiplex PCR Method for Simultaneous Detection of Infectious Laryngotracheitis Virus and *Ornithobacterium rhinotracheale*

**DOI:** 10.3390/vetsci10040272

**Published:** 2023-04-03

**Authors:** Van-Giap Nguyen, Thi-Bich-Phuong Cao, Van-Truong Le, Ha-Thai Truong, Thi-Thanh-Huong Chu, Huu-Anh Dang, Thi-Hoa Nguyen, Thi-Luyen Le, Thi-My-Le Huynh

**Affiliations:** 1 Department of Veterinary Microbiology and Infectious Diseases, Faculty of Veterinary Medicine, Vietnam National University of Agriculture (VNUA), Hanoi 100000, Vietnam; 2 Key Laboratory for Veterinary Biotechnology, Vietnam National University of Agriculture (VNUA), Hanoi 100000, Vietnam

**Keywords:** multiplex PCR, infectious laryngotracheitis virus, *Ornithobacterium rhinotracheale*

## Abstract

**Simple Summary:**

Infectious respiratory diseases in poultry can be induced by viruses, bacteria, mycoplasmas, and fungi in single or mixed infections. Multifactorial infections require multiplex screening for the pathogens of concern. To meet this demand, gel-based multiplex PCR assays have been regularly developed and widely applied to simultaneously detect ten common respiratory pathogens (viruses and bacteria) in chickens. However, to date, there are no available gel-based multiplex PCR assays for the co-detection of *Ornithobacterium rhinotracheale* and infectious laryngotracheitis virus. In this study, we successfully developed and applied a new multiplex PCR method to detect these two pathogens in field samples.

**Abstract:**

To date, many fluorescence- and gel-based multiplex polymerase chain reaction (PCR) assays have been developed for the simultaneous detection of multiple infectious agents of respiratory disease in poultry. However, PCR assays are not available for other important emerging respiratory bacteria, such as *Ornithobacterium rhinotracheale* (ORT). We aimed to fill this gap by establishing a new duplex PCR method for the simultaneous detection of infectious laryngotracheitis virus (ILTV) and ORT. Multiplex primer design software was used to select the compatible multiplex primer pairs. It was determined that an annealing temperature of 65 °C and an initial concentration of 2.5 pmol/µL for each primer set were the most suitable conditions for multiplex PCR. The assay was confirmed to be specific, as it only detected the target pathogens, even in the presence of six non-target agents. The limit of detection was up to 10^3^ copies/µL of template DNA for both ILTV and ORT. In the screening of 304 field samples, 23, 88, and 44 were positive for both ILTV and ORT, solely for ILTV, and solely ORT, respectively.

## 1. Introduction

Respiratory disease is regarded as one of the most important diseases affecting poultry and adversely affects animal health and welfare, as well as production, due to increased morbidity and mortality rates, reduced growth, and decreased egg production [1,2,3,4]. The infectious agents of respiratory diseases in poultry are diverse and include viruses, bacteria, mycoplasma, and fungi [5]. One the most well-known respiratory diseases of poultry is infectious laryngotracheitis (ILT) which caused by *Gallid herpesvirus type 1* (ILTV) [6]. ILTV has a large double-stranded DNA genome of about 152 kb [7]. Based on the whole-genome sequences, ILTV is divided into four clades (I-IV) [6]. In another system, based on the restriction fragment length polymorphism patterns of several selected genes, ILTV is defined into ten classes (1–10) [8,9]. Despite of varying in virulence, there is no link between the genetic classifications with the virulence of ILTV strains [10,11]. In contrast to ILT, Ornithobacteriosis is recently recognized as important emerging respiratory virus in poultry [12,13]. The causative agent, *Ornithobacterium rhinotracheale* (ORT), is a Gram-negative, pleomorphic bacteria of the genus *Ornithobacterium* [14]. To date, 18 serotypes (A to R) of ORT have been determined [14]. Of which, serotype A was the most prevalent one (94%) [15]; serotypes F, K, and M were “only occasionally isolated” [16]. It was acknowledged that different pathogenicity might exist between isolates [13]. However, it is still proposed that some serotypes (B, G, F, K, and M) may be less virulent than serotype A is [16,17].

Of the infection properties, respiratory pathogens can independently induce a disease; however, mixed infections are common [18]. Multifactorial infections affecting the respiratory system are further complicated due to an increasing number of novel microorganisms, which have been identified through metagenomic technology [1]. The circulation of multiple infectious agents requires multiplex screening for pathogens of concern. Currently, the multiplex polymerase chain reaction (PCR) assay is widely applied for detecting respiratory agents in poultry; some assays require a specialized method, such as capillary electrophoresis to analyze PCR products (e.g., in GeXP-multiplex PCR) [19] and fluorescence-based detection in high-throughput nanofluidic PCR platform [20] or multiplex real-time PCR [21]. Generally, the gel-based multiplex PCR assay is still widely applied. To date, ten respiratory pathogens (viruses and bacteria) have been targeted through the use of gel-based multiplex PCR, including avian influenza virus (AIV), infectious bronchitis virus (IBV), Newcastle disease virus (NDV), infectious laryngotracheitis virus (ILTV), fowl pox virus (FWPV), avian metapneumovirus (aMPV) and the bacterial species *Mycoplasma gallisepticum* (MG), *Mycoplasma synoviae* (MS), *Escherichia coli*, and *Pasteurella multocida* (PM) [22,23,24,25,26,27]. To the best of our knowledge, there are no available gel-based multiplex PCR methods developed for other important respiratory bacteria, such as *Avibacterium paragallinarum* (APG, previously known as *Haemophilus paragallinarum*) and *Ornithobacterium rhinotracheale* (ORT). To fill this gap, a duplex PCR was developed for the simultaneous detection of ILTV and ORT, one of the most well-known and important emerging respiratory pathogens [12,13]. 

## 2. Materials and Methods

### 2.1. Control Sample from Vaccines

DNA extracted from either live or inactivated vaccines was used as the control template. The following vaccines were used: (1) Medivac ILT (Medion), which contains a live attenuated ILTV strain; (2) Ornitin Triple (Phibro), an inactivated oil emulsion vaccine containing ORT serotypes A, B, and C; (3) Vaxsafe MG (Bioproperties), a live attenuated vaccine containing MG strain ts-11; (4) Vaxsafe MS (Bioproperties), a live attenuated vaccine containing MS- H strain; (5) Medivac Pox (Medion), which contains a live attenuated FWPV M92 strain; (6) Cevac corymune 4 K (Ceva), an inactivated aluminum hydroxide adjuvant vaccine combining APG serotypes A, B, and C. The reference strains of Avian pathogenic *Escherichia coli* (APEC), and *Pasteurella multocida* used in cross-reactivity tests were provided by colleagues in our department. The method of extracting DNA from vaccines was the same as that for the tissue samples described in Section 2.3.

### 2.2. Field Samples

From June 2021 to December 2022, 304 samples (organs of the respiratory systems of dead chickens) were obtained from different farms in 15 provinces in northern and central Vietnam. These samples were obtained from chicken flocks showing signs of respiratory disease and were submitted by farmers or field veterinarians for the purpose of diagnosis. Upon receipt of the samples, the respiratory organs (nasal turbinate, larynx, trachea, and lungs) were pooled, minced, and manually homogenized in a 10% suspension in phosphate-buffered saline (PBS) by grinding them using a mortar and pestle. The suspension was centrifuged at 4000 rpm for 5 min to collect the supernatant, which was used for total DNA extraction (described in Section 2.3).

### 2.3. DNA Extraction and Purification

Total DNA was manually extracted from proteinase K-digested samples using the phenol/chloroform extraction method, as previously described [28], with a slight modification. Briefly, (1) 200 µL of the sample (either 10% tissue suspension, reconstituted vaccine, or inactivated vaccine) was lysed in 500 µL of sucrose/proteinase K solution at 56 °C for 90 min. (2) Phase separation of DNA was performed by adding 200 µL of phenol-chloroform-isoamyl solution (25:24:1). (3) DNA was precipitated using 2-propanol at −20 °C for 15 min. (4) The DNA precipitate was then washed with 70% alcohol. (5) The DNA precipitate was dried and dissolved in 30 µL TE buffer (pH 8.0). Between steps (2) and (4), there was a centrifugation step at 12,000 rpm/15 min at 4 °C. Vigorous vortexing for 1 min was performed in steps (1) and (2).

### 2.4. Multiplex Primer Design

From a review of the literature, a list was compiled of genes that had been used in PCR-based assays to detect ILTV and ORT. Target genes for primer design were selected: thymidine kinase (TK) gene (MN073052) for ILTV [29] and the Or01 gene (dihydrolipoamide acetyltransferase) (AJ748732) for ORT [30].

MPprimer-1.4 software [31] was used to design multiplex primers with the following identical input criteria for ILTV and ORT: PRIMER_OPT_SIZE = 22, PRIMER_MAX_SIZE = 27, PRIMER_MIN_SIZE = 18, PRIMER_OPT_TM = 60.0, PRIMER_MAX_TM = 63.0, PRIMER_MIN_TM = 57.0, PRIMER_OPT_GC_PERCENT = 50.0, PRIMER_MAX_GC = 60.0, and PRIMER_MIN_GC = 40.0. 

The input option for amplicon length (PRIMER_PRODUCT_SIZE_RANGE=) was adjusted in the preferred range from 200 to 300 bp for ILTV and from 400 to 500 bp for ORT. The multiplex primers used in this study are listed in Table 1. Primers were synthesized by PhuSa Genomics (Can Tho, Vietnam). 

### 2.5. Evaluation of Primers

The specificity of the primer set was checked using two methods. First, primer sequences (Table 1) were input into the BLAST tool (https://blast.ncbi.nlm.nih.gov/Blast.cgi, accessed on 1 September 2022) to search for highly similar sequences in the standard database of nucleotide collections (nr/nt). A primer was considered specific if the complete sequence of the primer (100% query coverage) produced significant alignments with the corresponding pathogen (percent identity ≥ 95%). The specificity of the primers was further tested by multiplex PCR using different templates and combinations of DNA viruses, bacteria, and mycoplasmas commonly involved in respiratory diseases in chickens (Table 2, Appendix A Table A2). The primer set was specific if only the template of the corresponding target (ILTV and ORT) showed a band of the expected size. The profile of the PCR is given in Section 2.6.

### 2.6. Optimizing Multiplex PCR

A commercial PCR master mix (2X H-Star Taq PCR Master Mix 2, HS303-40h, BioFACT, Daejeon, Korea) was used for nucleic acid amplification. Thus, optimization of the reaction was focused first on the annealing temperature and second on the primer concentration. Additionally, simplified optimization was achieved by performing experiments directly using multiplex PCR settings. The most suitable annealing temperature (°C) was determined from the following ones: 65.0, 64.5, 63.3, 61.4, 58.9, 57.1, 55.8, and 55.0 °C (row A–H of a gradient thermal cycler, iCycler, Bio-Rad, Hercules, CA, USA). The optimal primer concentration was determined after evaluating the appearance and density of the product bands on the agarose gel at different concentrations, as presented in Table 3.

The multiplex PCR reaction contained 5 µL of 2X PCR master mix, 1.0 µL aliquot of each primer mix (2.5 pmol/µL each of 5ILTV.233F/R and 6ORT.467F/R), 1 µL DNA template, and distilled water up to 10 µL. The basic thermal profile of the multiplex PCR was 95 °C for 15 min, followed by 50 cycles of 95 °C/20 s, 65 °C/30 s (except for the annealing temperature optimization experiment), 72 °C/50 s, and held at 25 °C until completion. The PCR products were mixed with Loading Buffer 6X SyBR Green I (P-LBS6X, PhuSa Genomics, Vietnam) and analyzed through agarose gel electrophoresis. The results were visualized using GelPic100 Box (PhuSa Genomics, Vietnam). 

### 2.7. Determining the Limit of Detection of the Multiplex PCR

The limit of detection (LOD) of the multiplex reaction was determined based on a standard curve. The standard template was prepared as follows. Two large (874 bp) fragments of the TK gene and 1315 bp of the Or01 gene were amplified using the primer sets shown in Appendix A Table A1. The large fragments contained binding sites for 5ILTV.233F/R and 6ORT.467F/R primers. The 874 bp and 1315 bp PCR fragments were excised from the agarose gel and purified using GeneJET Gel Extraction (K0691, Thermo Fisher Scientific, Waltham, MA, USA). The DNA concentration of the purified fragments (37 ng/µL for 874 bp fragments; 31 ng/µL for 1315 bp fragments) was determined using a QuickDrop Micro-Volume Spectrophotometer (Molecular Devices). The template copy number was calculated using the following equation: template copies/µL = [amount of DNA (ng/µL) × (6.022 × 10^23^)]/[fragment length (bp) × 10^9^ × 650], which is available from http://cels.uri.edu/gsc/cndna.html (accessed on 1 October 2022). Purified PCR fragments with known copy number (3.92 × 10^10^ for 874 bp fragment; 2.18 × 10^10^ for 1315 bp fragment) were serially diluted ten-fold from 10^−1^ to 10^−10^ to create standard curves. The LOD of the multiplex PCR assay was determined under optimized conditions of the annealing temperature and primer concentration. 

### 2.8. Detection of ILTV and ORT in Field Samples by Multiplex PCR Assay

To evaluate the diagnostic value of this newly developed multiplex PCR method, 304 field samples were screened. To further confirm the detection results, PCR amplicons of positive ILTV and ORT samples were randomly selected for Sanger sequencing (1st BASE, Selangor, Malaysia). Using the BLAST tool, the 233 bp and 467 bp amplicons obtained were checked to determine whether they were identical to ILTV and ORT, respectively. 

## 3. Results

### 3.1. Optimization of Multiplex PCR

**Figure 1:**
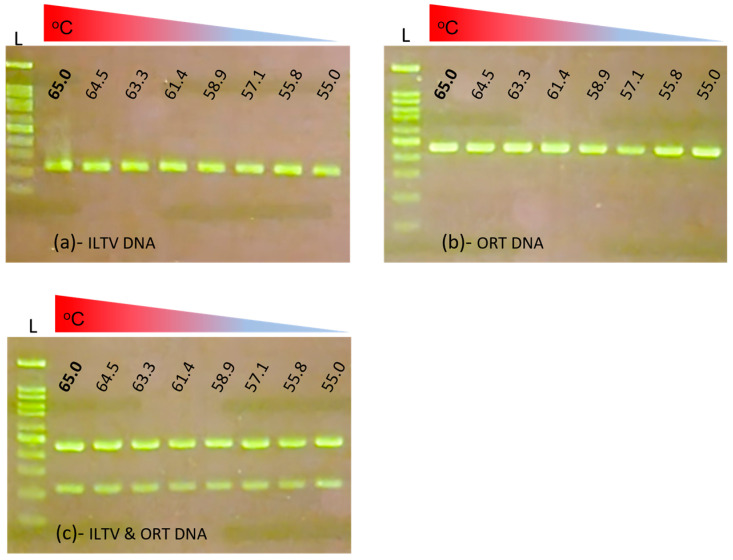
Optimization of annealing temperature. Multiplex PCR reactions were performed with single DNA template of ILTV (**a**) or ORT (**b**) and with double-DNA of ILTV and ORT (**c**). One hundred bp DNA ladder (L); the lowest band was on hundred bp and the brightest band was five hundred bp.

The first optimization result was the optimal annealing temperature of the multiplex primers (Figure 1). 

**Figure 2:**
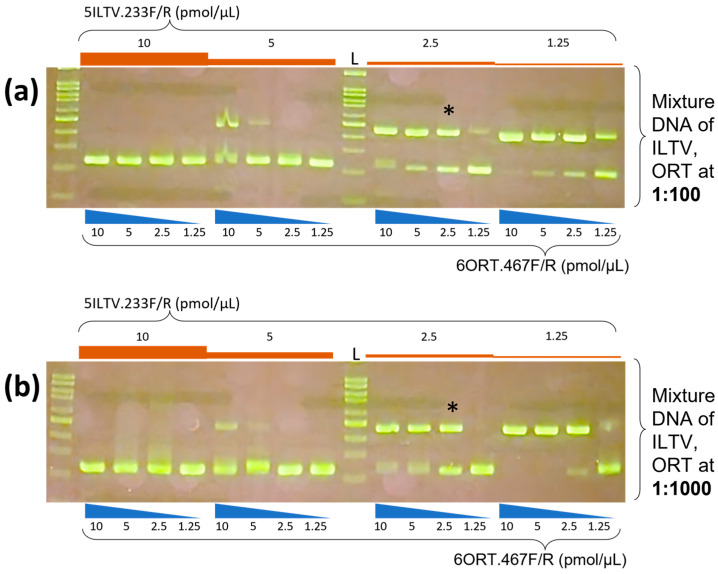

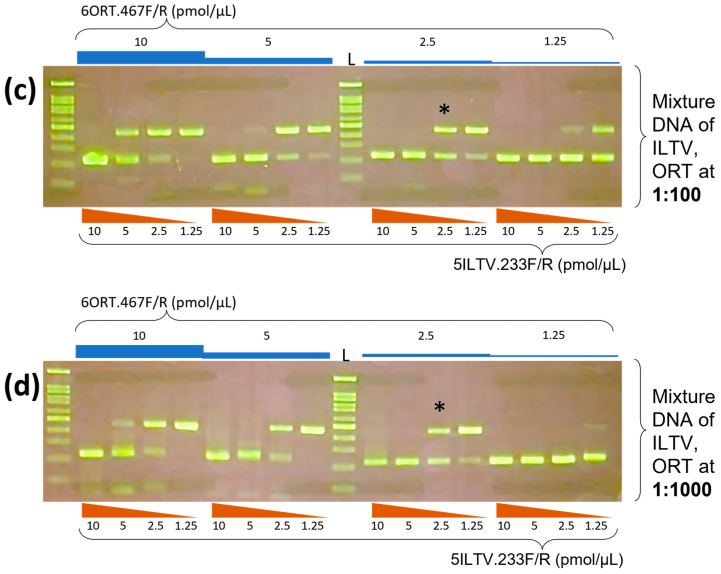
Optimization of primer concentration. In total, 16 combinations of different concentration of specific primers for ILTV and ORT were evaluated. The range of initial concentration for each primer set was from 10 to 1.25 pmol/µL. (**a**,**b**) Results of multiplex PCR with variable concentration of 6ORT.467F/R at each fixed concentration of 5ILTV.233F/R. (**c**,**d**) Results of multiplex PCR with variable concentration of 5ILTV.233F/R at each fixed concentration of 6ORT.467F/R. The multiplex PCR reactions were performed at optimal annealing temperature and with two dilutions of mixture template DNA: 100 bp DNA ladder (L); the lowest band was 100 bp. * indicates the optimal concentration.

Generally, in the continuous range from 55 to 65 °C, two specific bands were observed. Based on the brightness of the target amplification bands, a maximum annealing temperature of 65 °C was chosen as the most suitable one for the multiplex reaction. The results of the optimization of the primer concentration are shown in Figure 2. 

Not all combinations of primer concentrations generated specific double bands. At either a 10-fold difference in dilutions of the mixture template DNA, as shown in Figure 2a,b, if the initial concentration of 5ILTV.233F/R was from 2.5–1.25 pmol/µL, the primer 6ORT.467F/R in the concentration range of 10–2.5 pmol/µL consistently generated the specific 467 bp amplicon of ORT. Similarly, as shown in Figure 2c,d, if the initial concentration of 6ORT.467F/R was 2.5–1.25 pmol/µL, the primer 5ILTV.233F/R in the concentration range of 10–1.25 pmol/µL consistently generated the specific 233 bp amplicon of ILTV. These results suggest that the initial 2.5–1.25 pmol/µL concentration of each 6ORT.467F/R and 5ILTV.233F/R primer set allowed the significant co-amplification of 233 and 467 amplicons. However, for the most balanced amplification two targets with strong, specific bands, and little or no non-specific product, the initial 2.5 pmol/µL for each primer set (* in Figure 2) was determined as the optimal concentration for this multiplex PCR.

### 3.2. Specificity and Limit of Detection of Multiplex PCR

**Figure 3:**
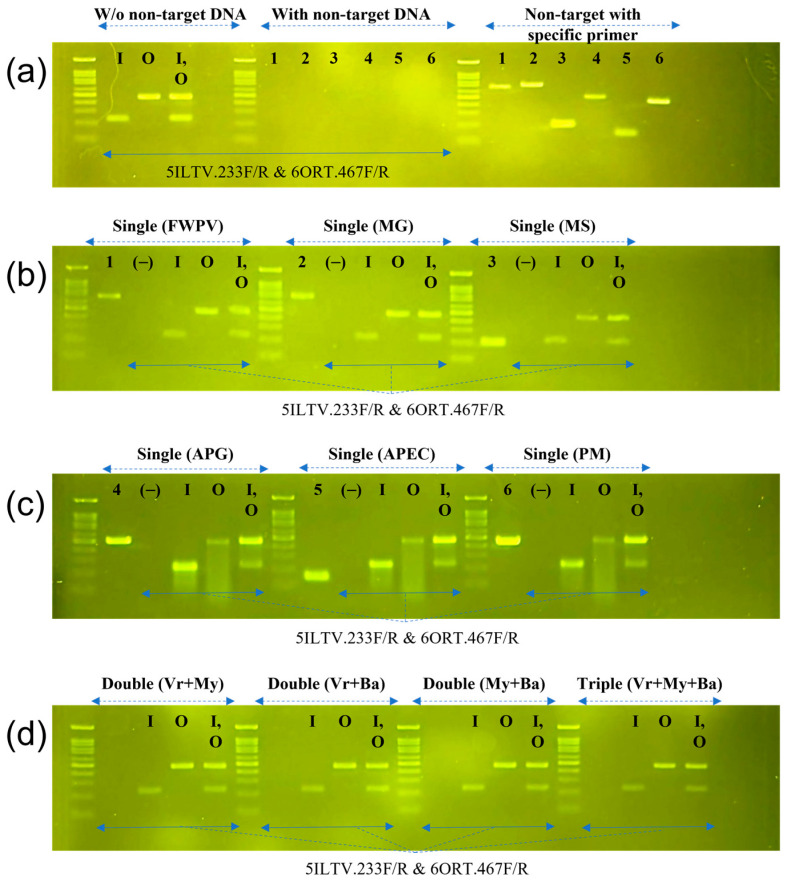
Agarose gel electrophoresis showing the specificity of multiplex PCR. (**a**) Multiplex PCR was performed solely with target DNA of ILTV (I), ORT (O) or both (I, O); and with mixture of non-target DNA of FWPV (1), MG (2), MS (3), APG (4), APEC (5), or PM (6). (**b**,**c**) The multiplex with target DNA in the presence of single non-target DNA. Distilled water was used as negative control (−). (**d**) Six non-target pathogens are divided into three groups of virus (Vr: FWPV), mycoplasma (My: MG, MS), and bacteria (Ba: APG, APEC, and PM). The reactions with target DNA were then performed in the presence double DNA from either Vr + My, Vr + Ba, or My + Ba, as well as in the presence of triple DNA from Vr + My + Ba.

The specificity of the multiplex PCR was evaluated using a single sample or a mixture of DNA from six common respiratory pathogens in chickens (FWPV, MG, MS, APG, APEC, and PM) (Figure 3). 

As shown in Figure 3a (lanes 1–6), in all cases where no ILTV or ORT DNA was present, a specific band was absent. In contrast, the presence of ILTV, ORT, or both ILTV and ORT in the mixture with the other DNA (Figure 3b–d), no matter the number of combination of non-target DNAs, generated only a specific single or duplex band (lanes I, O, and I,O). These results indicate that the multiplex primer set was specific for ILTV and ORT. 

**Figure 4:**
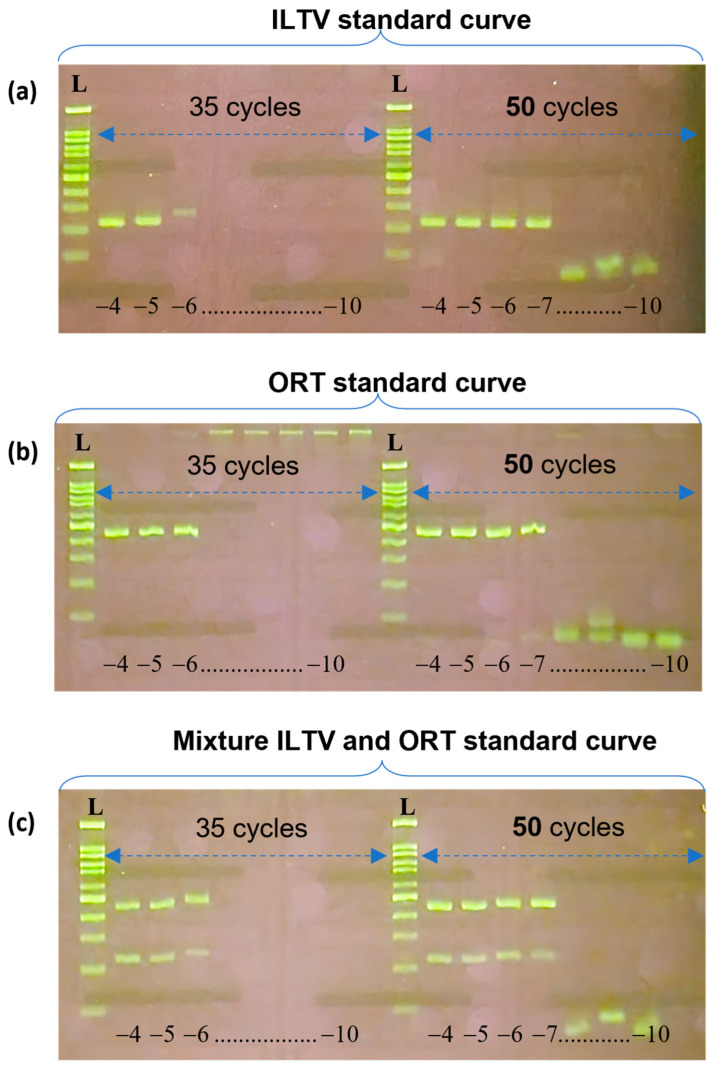
Agarose gel electrophoresis showing the limit of detection of multiplex PCR. (**a**–**c**) Limit of detection of multiplex PCR was determined after 35 and 50 thermal cycles. To save reagents, the starting dilution used was from 10^−4^ (−4) to 10^−10^ (−10), with the copy number equivalent to 3.92 × 10^6^ for ILTV and 2.18 × 10^6^ for ORT. On hundred bp DNA ladder (L); the lowest band was one hundred bp, and the brightest band was five hundred bp.

Concerning the limit of detection, in both the single sample (Figure 4a,b) or the mixture (Figure 4c), from the standard curve, ILTV is detectable at the dilution of 10^−7^ (equal to 3.92 × 10^3^ copies/µL), and ORT is detectable at the dilution of 10^−7^ (equal to 2.18 × 10^3^ copies/µL). It was noted that the number of thermal cycles affected the results. Multiplex PCR with 35 cycles was 10 times less sensitive than that with 50 cycles. 

### 3.3. Application of Multiplex PCR on Field Samples

**Figure 5:**
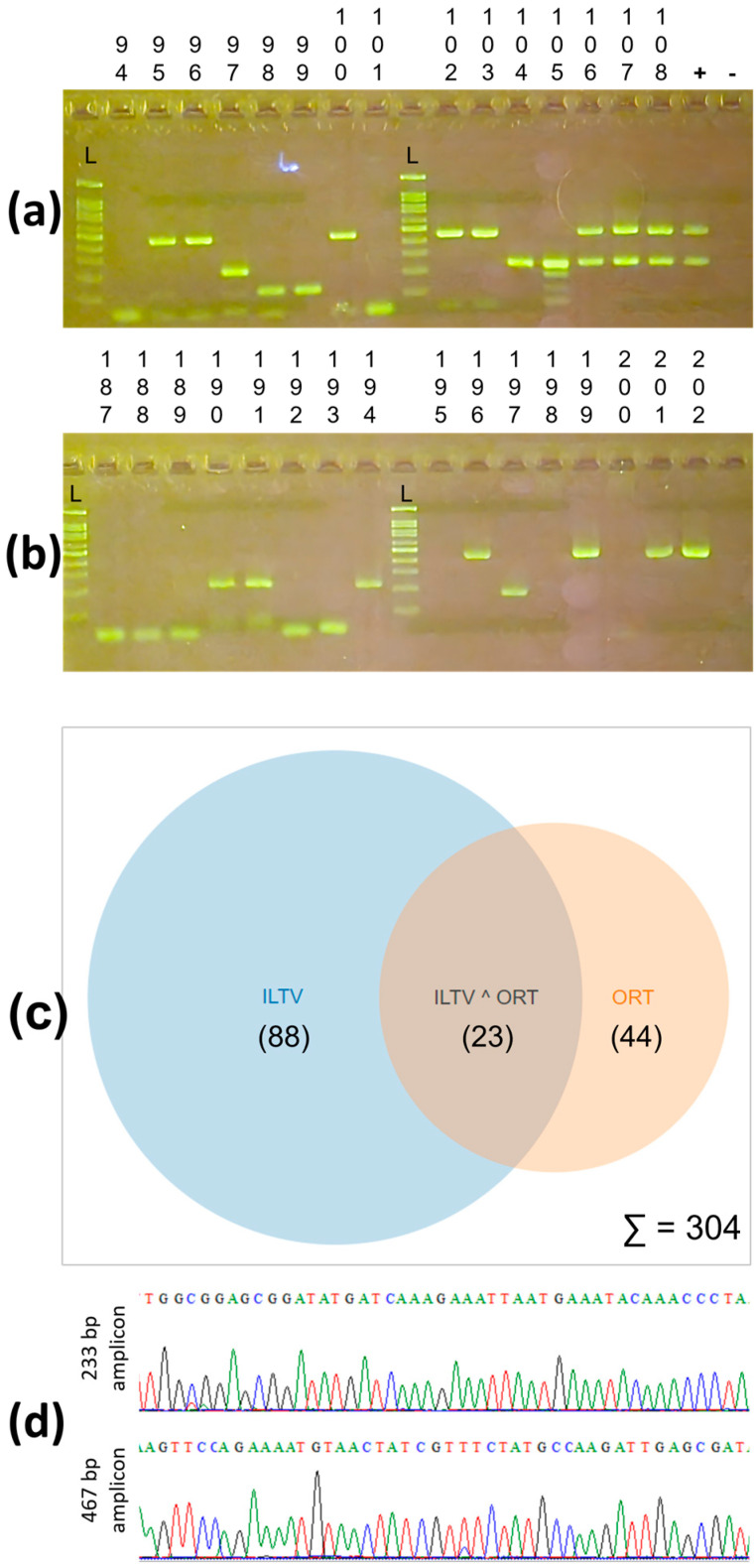
The diagnostic results of the multiplex PCR. (**a**,**b**) Agarose electrophoresis of field samples. One hundred bp DNA ladder (L); the lowest band was one hundred bp, and the brightest band was five hundred bp. (**c**) Venn diagram of the number of positive samples for ILTV, ORT, and both pathogens. (**d**) Partial results of sequencing chromatograms of the 233 and 467 bp amplicons.

A clear and specific band, indicating the expected size for single (ILTV or ORT) and dual (ILTV and ORT) positive samples can be seen in Figure 5a,b. On a few occasions, such as the samples in lanes 98, 99, and 105, non-specific bands were observed. However, they were different in terms of the size of the expected target bands, and this should not interfere with the result’s interpretation. Of the 304 samples, 23 were positive for both ILTV and ORT, and 88 and 44 tested positive solely for ILTV and ORT, respectively (Figure 5c). The overall positivity rates for ILTV and ORT were 36.5% (111/304) and 22.0% (67/304), respectively. Ten and six amplicons from ILTV and ORT (~1% of the 155 positive samples) were randomly selected and subjected to Sanger sequencing (Figure 5d). BLAST results (not shown) confirmed that the sequences of the 233 and 467 bp PCR products were identical to those of ILTV and ORT, respectively.

## 4. Discussion

### 4.1. Selection of the Target Pathogens and Detection Method

There are currently several agarose-based detection multiplex PCR assays available for a mixed infection of ILTV, FWPV, and reticuloendotheliosis virus [32]; ILTV, AIV, NDV, and IBV [24]; ILTV, IBV, AIV, NDV, MG, and MS [23]. However, a multiplex method for the combined detection of ORT and other respiratory pathogens is still lacking. In this study, RNA viruses were not chosen to develop a multiplex PCR with ORT because they required an additional step of the conversion of RNA into complementary DNA. To further the development of more complex multiplex PCR methods, we have focused on a duplex PCR for ILTV and ORT. Another reason was that ORT is acknowledged as an increasingly important pathogen in poultry [12], and ILTV remains an important pathogen of upper respiratory disease in chicken [33].

With respect to infectious diseases of poultry, the multiplex PCR assay is one technique that has been widely applied for various purposes, such as: pathotyping, serotyping, toxinotyping [34,35,36,37], simultaneous pathogen detection [23,26,38], and differentiating field and vaccine strains [39]. In addition to the increasingly popular fluorescence-based method of visualizing PCR amplifications [4,5], gel-based methods are still widely used, especially in less well-equipped laboratories. The results of this study contribute to the development of a new multiplex gel-based PCR for two important respiratory pathogens in chickens (ILTV and ORT). The current method requires the prior extraction and purification of DNA. Currently, commercial direct PCR amplification kits without nucleic acid extraction are available. Thus, future research is warranted to adapt the conventional multiplex PCR kits to a direct multiplex PCR format. 

### 4.2. Multiplex Primer Design and Validation

To develop a new multiplex PCR reaction, it might be possible to use software, such as MultiPLX [40], to automatically group existing primers for multiplexed PCR. However, after several laboratory trials, we experienced difficulty in achieving satisfactory amplification (data not shown). Thus, we decided to use a specialized program to achieve the design of a reliable multiplex PCR primer. To design target-specific primers, we first reviewed the literature to identify the specific genes that had been previously used in PCR assays to detect ILTV and ORT. 

At least three ILTV genes have been successfully used as templates for primer selection: infected cell protein 4 (ICP4) [41,42], thymidine kinase (TK) [19,29,43], and glycoprotein E (gE) [44]. In the present study, we preferred the TK gene as it is a major gene related to the virulence of ILTV [45], with a higher percent of nucleotide identity (98–100%) than those of others (99–100% for gE, and 89–100% for ICP4). Further genetic analysis with reference strains of four ILTV clades (Supplementary Figure S1a) revealed that the newly designed ILTV primers matched the majority of homologous sequences available in GenBank (171/172 for 5ILTV.233F; 231/235 for 5ILTV.233R) and were able to detect all the clades.

In the case of ORT, the 16S rRNA gene was the gene most targeted one in published studies [46,47,48]; although other genes have also been used: gyrA [20], rpoB [49], or the Or01 gene (dihydrolipoamide acetyltransferase) [17,30]. We selected the Or01 gene, which encodes cross-reactive antigens between ORT strains [17]. A limitation of this study while evaluating the ORT specific primer was that there are no publicly available Or01 sequences for all 18 ORT serotypes; thus, it was only possible to compare the primer sequences with a limited number of serotypes (A, G, K, F, and M). As shown in Supplementary Figure S1b, at the first five nucleotides of the 3′ end of each primer, the forward primer (6ORT.467F) contained a mismatch with serotype M of ORT. Theoretically, that terminal mismatch will inhibit the PCR amplification process [50]. Among the 18 (A–R) serotypes of ORT [12], serotype A was the most prevalent one (94%) [15]. Additionally, it was noted that serotypes F, K, and M are “only occasionally isolated” from birds all over the world [16]. As a result, the primer set for ORT (Table 1) is believed to be suitable for most ORT strains. However, this study contained another shortage when it was unable to validate the duplex PCR on a collection of well-defined ORT reference serotypes.

With respect to LOD, the method developed in this study was able to detect up to 10^3^ copies/µL of template DNA for both ILTV and ORT (Figure 4a). This LOD is comparable to those reported for some multiplex PCR methods for detecting pathogens in farm animals [37,51,52]. In a previous multiplex PCR assay, the detection limit of ILTV was reported to be 100 pg [24]. Taking the average size (153,196 nucleotides) of the complete genome of 85 ILTV strains in GenBank, 100 pg of ILTV genome DNA would be equal to 6.05 × 10^5^ copies (conversion tool at http://cels.uri.edu/gsc/cndna.html, accessed on 1 October 2022). Therefore, the method developed in the current study is more sensitive to ILTV detection. To the best of our knowledge, there are no available direct LOD results for ORT. According to the linear relationship between the template amount and Ct value [47], at a Ct of 32.81, the copy number was calculated as 3.65 × 10^4^. At this concentration, the widely applied conventional PCR for ORT using OR16S-F1/OR16S-R1 primers was negative (Table 2 of reference [47]). Therefore, it is safe to conclude that the multiplex PCR developed in this study was at least 10 times more sensitive. These results show that the newly developed multiplex PCR assay is sufficiently sensitive for diagnostic purposes.

### 4.3. Application of the Newly Developed Multiplex PCR

The multiplex PCR assay was used to screen a large number of field samples collected from flocks showing signs of respiratory disease. The results (Figure 5) show that the method was effective, given that we were able to detect single and mixed infections of ILTV and ORT. The positive rates were 36.5% (111/304) for ILTV, 22.0% (67/304) for ORT, and 7.6% (23/304) for concurrent ILTV and ORT infections. In Vietnam, there are many common respiratory pathogens in circulation in chicken flocks [53,54,55]; however, there are only a few reports available on their prevalence. Consequently, it was not possible to compare the detection rates of ILTV. The ORT detection rate reported in this study was somewhat higher than that in previous studies, which varied from 9% to 13% [53,55]. Considering the differences in sampling designs, one possible explanation might be related to the use of the same specific ORT primer (OR16S-F1/OR16S-R1) [46] used in previous studies [53,55]. Although OR16S-F1/OR16S-R1 has been applied worldwide, it has been pointed out that it is not the best method for diagnosis, considering that it has failed to detect ORT in certain circumstances [47]. Moreover, the method developed in the current study was shown to have better sensitivity for ORT. This might explain the higher ORT-positive rates observed in this study. However, in future studies, it will be necessary to compare the diagnostic performance of this novel multiplex PCR assay with other related assays. 

## 5. Conclusions

In this study, we successfully designed and optimized the working conditions of a new multiplex PCR for the simultaneous detection of ILTV and ORT. The method was demonstrated to be specific, sensitive, and capable of recognizing both single and mixed ILTV and ORT infections from field samples. The results of our study confirm that this multiplex PCR is another useful tool for detecting respiratory pathogens in chickens.

## Figures and Tables

**Table 1:** Sequences of primers designed in this study.

Forward/Reverse	Pathogen	Sequence (5′-3′)	Expected Size (bp)
5ILTV.233F	ILTV	CCGACTTTCGCCGCGTTGTACT	233
/5ILTV.233R		ACGAGACGCCTCCCGACATTCA	
6ORT.467F	ORT	AGCGGTGGAGGTGCTAGCCAAT	467
/6ORT.467R		GCAGCAGGCGCTGGAGTTTCTT	

**Table 2:** Test to validate primer specificity.

Experiment	Template DNA		Primer Mix	Expected Result	
	Non-Target ^1^	Target ^2^		ILTV	ORT
1	None	ILTV	5ILTV.233F /5ILTV.233R and 6ORT.467F /6ORT.467R	+	-
2	None	ORT		-	+
3	None	ILTV; ORT.		+	+
4	None	Water		-	-
5	Single	None		-	-
6	Single	ILTV		+	-
7	Single	ORT		-	+
8	Single	ILTV; ORT.		+	+
9	Double	ILTV		+	-
10	Double	ORT		-	+
11	Double	ILTV; ORT.		+	+
12	Triple	ILTV		+	-
13	Triple	ORT		-	+
14	Triple	ILTV; ORT.		+	+

^1^ There are six non-target pathogens: fowl pox virus (FWPV), *Mycoplasma gallisepticum* (MG), *Mycoplasma synoviae* (MS), *Avibacterium paragallinarum* (APG), Avian pathogenic *Escherichia coli* (APEC), and *Pasteurella multocida* (PM). They are divided into three groups of viruses (FWPV), mycoplasma (MG and MS), and bacteria (APG, APEC, and PM). Single DNA contains either FWPV, MG, MS, APG, APEC, or PM mixes with the target DNA(s). Double DNA contains either virus mycoplasma, virus bacteria, or mycoplasma bacteria mixes with the target DNA(s). Triple DNA contains virus mycoplasma–bacteria mixes with target DNA(s). ^2^ Extracted DNA from infectious laryngotracheitis virus (ILTV), *Ornithobacterium rhinotracheale* (ORT).

**Table 3:** Experiment design to determine the optimal combination of concentrations.

		5ILTV.233F/5ILTV.233R Mix (pmol/µL)			
		(10)	(5)	(2.5)	(1.25)
**6ORT.467F/6ORT.467R Mix (pmol/µL)**	(10)	1	2	3	4
	(5)	5	6	7	8
	(2.5)	9	10	11	12
	(1.25)	13	14	15	16

## Data Availability

The data supporting the findings of this study are included in the Supplementary Materials.

## Supplementary Materials

The following supporting information can be downloaded at: https://www.mdpi.com/article/10.3390/vetsci10040272/s1, Figure S1: Supplementary Figure S1 alignment of primers.

## Appendix A

**Table A1:** List of primers used to prepare standard curve for ILTV and ORT.

Forward/Reverse ^1^	Gene	Sequence (5′-3′)	Reference
TK_792F	TK	TGGCAACGGATTCCGGCA	[29]
TK_673R		TTGCAAATAGCGTCTGGTCGATTGAA	[43]
Or01_1607F	Or01	GCTGAAATTATAAAAATGCC	[17]
Or01_1607R		CACAAGCATAGACATTGG	[17]
Or01_1315F		ACATCAGCGGTTTGGTAAGC	This study
Or01_1315R		AGTACTTCCAGTTGCTCCGT	This study

^1^ The primers were re-named in this study.

For the TK fragment, conventional PCR was performed using the primers TK_792F/TK_673R. For the Or01 fragment, the first PCR was performed using primers Or01_1607F/Or01_1607R. The PCR product was then used as a template for the second PCR that was performed using primers Or01_1315F/Or01_1315R. The PCR master mix was the same as that described in Section 2.6. In all cases, the initial primer concentration was 10 pmol/µL. The thermal profile was 95 °C for 15 min, followed by 50 cycles of 95 °C/20 s, 52 °C/30 s, 72 °C/120 s, and it was held at 25 °C until we stopped.

**Table A2:** List of primers used to detect pathogens in the specificity test.

Forward/Reverse ^1^	Pathogen	Sequence (5′-3′)	Expected Size (bp)	Reference
Pox.Po681R	FWPV	CGCCGCATCATCTACTTATC	681	[56]
Pox.Po681F		CCACACAGCGCCATTCATTA		
TspE4C2.152F	E. coli	CACTATTCGTAAGGTCATCC	152	[57]
TspE4C2.152R		AGTTTATCGCTGCGGGTCGC		
PM.460F	PM	GCTGTAAACGAACTCGCCAC	460	[58]
PM.460R		ATCCGCTATTTACCCAGTGG		
MS.207F	MS	GAGAAGCAAAATAGTGATATCA	202	[59]
MS.207R		CAGTCGTCTCCGAAGTTAACAA		
MG.732F	MG	GGATCCCATCTCGACCACGAGAAAA	732	[60]
MG.732R		CTTTCAATCAGTGAGTAACTGATGA		
APG.500F	APG	CAATGTCGATCCTGGTACAATGAG	500	[61]
APG.500R		CAAGGTATCGATCGTCTCTCTACT		

^1^ The primers were re-named in this study.

## References

[1] Rajeoni A.H., Ghalyanchilangeroudi A., Khalesi B., Madadi M.S., Hosseini H. (2021). The tracheal virome of broiler chickens with respiratory disease complex in Iran: The metagenomics study. Iran. J. Microbiol..

[2] Sid H., Benachour K., Rautenschlein S. (2015). Co-infection with multiple respiratory pathogens contributes to increased mortality rates in Algerian poultry flocks. Avian Dis..

[3] Roussan D.A., Haddad R., Khawaldeh G. (2008). Molecular survey of avian respiratory pathogens in commercial broiler chicken flocks with respiratory diseases in Jordan. Poult. Sci..

[4] Hassan M.S.H., Abdul-Careem M.F. (2020). Avian viruses that impact table egg production. Animals.

[5] Kannaki T.R., Priyanka E., Haunshi S., Subbiah M. (2021). A systematic review and meta-analysis on global prevalence of infectious diseases in backyard chicken in the recent two decades. Indian J. Poult. Sci..

[6] García M., Spatz S. (2020). Infectious Laryngotracheitis. Diseases of Poultry.

[7] Lee S.W., Markham P.F., Markham J.F., Petermann I., Noormohammadi A.H., Browning G.F., Ficorilli N.P., Hartley C.A., Devlin J.M. (2011). First complete genome sequence of infectious laryngotracheitis virus. BMC Genom..

[8] Blacker H.P., Kirkpatrick N.C., Rubite A., O’Rourke D., Noormohammadi A.H. (2011). Epidemiology of recent outbreaks of infectious laryngotracheitis in poultry in Australia. Aust. Vet. J..

[9] Sabir A.J., Olaogun O.M., O’Rourke D., Fakhri O., Coppo M.J.C., Devlin J.M., Konsak-Ilievski B., Noormohammadi A.H. (2020). Full genomic characterisation of an emerging infectious laryngotracheitis virus class 7b from Australia linked to a vaccine strain revealed its identity. Infect. Genet. Evol. J. Mol. Epidemiol. Evol. Genet. Infect. Dis..

[10] Kirkpatrick N.C., Mahmoudian A., O’Rourke D., Noormohammadi A.H. (2006). Differentiation of infectious laryngotracheitis virus isolates by restriction fragment length polymorphic analysis of polymerase chain reaction products amplified from multiple genes. Avian Dis..

[11] Piccirillo A., Lavezzo E., Niero G., Moreno A., Massi P., Franchin E., Toppo S., Salata C., Palù G. (2016). Full Genome Sequence-Based Comparative Study of Wild-Type and Vaccine Strains of Infectious Laryngotracheitis Virus from Italy. PLoS ONE.

[12] El-Ghany W.A.A. (2021). An updated comprehensive review on ornithobacteriosis: A worldwide emerging avian respiratory disease. Open Vet. J..

[13] van Veen L., van Empel P., Fabri T. (2000). *Ornithobacterium rhinotracheale*, a Primary Pathogen in Broilers. Avian Dis..

[14] Boulianne M., Blackall P.J., Hofacre C.L., Ruiz J.A., Sandhu T.S., Hafez H.M., Chin R.P., Register K.B., Jackwood M.W. (2020). Pasteurellosis and Other Respiratory Bacterial Infections. Diseases of Poultry.

[15] Van Empel P., van den Bosch H., Loeffen P., Storm P. (1997). Identification and serotyping of *Ornithobacterium rhinotracheale*. J. Clin. Microbiol..

[16] Schuijffel D.F., Van Empel P.C., Segers R.P., Van Putten J.P., Nuijten P.J. (2006). Vaccine potential of recombinant *Ornithobacterium rhinotracheale* antigens. Vaccine.

[17] Schuijffel D.F., van Empel P.C., Pennings A.M., van Putten J.P., Nuijten P.J. (2005). Successful selection of cross-protective vaccine candidates for *Ornithobacterium rhinotracheale* infection. Infect. Immun..

[18] Abdelaziz A.M., Mohamed M.H.A., Fayez M.M., Al-Marri T., Qasim I., Al-Amer A.A. (2019). Molecular survey and interaction of common respiratory pathogens in chicken flocks (field perspective). Vet. World.

[19] Xie Z., Luo S., Xie L., Liu J., Pang Y., Deng X., Xie Z., Fan Q., Khan M.I. (2014). Simultaneous typing of nine avian respiratory pathogens using a novel GeXP analyzer-based multiplex PCR assay. J. Virol. Methods.

[20] Croville G., Foret C., Heuillard P., Senet A., Delpont M., Mouahid M., Ducatez M.F., Kichou F., Guerin J.-L. (2018). Disclosing respiratory co-infections: A broad-range panel assay for avian respiratory pathogens on a nanofluidic PCR platform. Avian Pathol..

[21] Laamiri N., Aouini R., Marnissi B., Ghram A., Hmila I. (2018). A multiplex real-time RT-PCR for simultaneous detection of four most common avian respiratory viruses. Virology.

[22] Saba Shirvan A., Mardani K. (2014). Molecular detection of infectious bronchitis and Newcastle disease viruses in broiler chickens with respiratory signs using duplex RT-PCR. J. Vet. Res. Forum.

[23] Pang Y., Wang H., Girshick T., Xie Z., Khan M.I. (2002). Development and application of a multiplex polymerase chain reaction for avian respiratory agents. Avian Dis..

[24] Rashid S., Naeem K., Ahmed Z., Saddique N., Abbas M.A., Malik S.A. (2009). Multiplex polymerase chain reaction for the detection and differentiation of avian influenza viruses and other poultry respiratory pathogens. Poult. Sci..

[25] Nguyen T.T., Kwon H.J., Kim I.H., Hong S.M., Seong W.J., Jang J.W., Kim J.H. (2013). Multiplex nested RT-PCR for detecting avian influenza virus, infectious bronchitis virus and Newcastle disease virus. J. Virol. Methods.

[26] Wang Z., Zuo J., Gong J., Hu J., Jiang W., Mi R., Huang Y., Chen Z., Phouthapane V., Qi K. (2019). Development of a multiplex PCR assay for the simultaneous and rapid detection of six pathogenic bacteria in poultry. AMB Express.

[27] Malik Y.S., Patnayak D.P., Goyal S.M. (2004). Detection of three avian respiratory viruses by single-tube multiplex reverse transcription-polymerase chain reaction assay. J. Vet. Diagn. Investig..

[28] Yang J.S., Song D.S., Kim S.Y., Lyoo K.S., Park B.K. (2003). Detection of porcine circovirus type 2 in feces of pigs with or without enteric disease by polymerase chain reaction. J. Vet. Diagn. Investig..

[29] Beltrão N., Egochega R.F., Furian T., Rodenbusch C., Fallavena L.C.B., Pasquali G., Canal C. (2012). A sensitive nested-polymerase chain reaction protocol to detect infectious laryngotracheitis virus. Acta Sci. Vet..

[30] Numee S., Hauck R., Hafez H.M. (2012). Detection and typing of Ornithobacterium rhinotracheale from German poultry flocks. Avian Dis..

[31] Shen Z., Qu W., Wang W., Lu Y., Wu Y., Li Z., Hang X., Wang X., Zhao D., Zhang C. (2010). MPprimer: A program for reliable multiplex PCR primer design. BMC Bioinform..

[32] Song H., Kim H., Kim S., Kwon Y., Kim H. (2021). Research Note: Simultaneous detection of infectious laryngotracheitis virus, fowlpox virus, and reticuloendotheliosis virus in chicken specimens. Poult. Sci..

[33] Gowthaman V., Kumar S., Koul M., Dave U., Murthy T., Munuswamy P., Tiwari R., Karthik K., Dhama K., Michalak I. (2020). Infectious laryngotracheitis: Etiology, epidemiology, pathobiology, and advances in diagnosis and control—A comprehensive review. Vet. Q..

[34] Heikinheimo A., Korkeala H. (2005). Multiplex PCR assay for toxinotyping Clostridium perfringens isolates obtained from Finnish broiler chickens. Lett. Appl. Microbiol..

[35] O’Regan E., McCabe E., Burgess C., McGuinness S., Barry T., Duffy G., Whyte P., Fanning S. (2008). Development of a real-time multiplex PCR assay for the detection of multiple *Salmonella* serotypes in chicken samples. BMC Microbiol..

[36] Tahir M.S., Mehmood D., Sultan A.U., Saeed M.H., Khan A.R., Ansari F., Salman M.M., Majeed K.A. (2016). A modified strategy of multiplex RT-PCR for simultaneous detection of H5, H7, and H9 subtypes of avian influenza virus based on common forward oligo. J. Appl. Poult. Res..

[37] Gao Q., Yun B., Wang Q., Jiang L., Zhu H., Gao Y., Qin L., Wang Y., Qi X., Gao H. (2014). Development and Application of a Multiplex PCR Method for Rapid Differential Detection of Subgroup A, B, and J Avian Leukosis Viruses. J. Clin. Microbiol..

[38] Caterina K.M., Frasca S., Girshick T., Khan M.I. (2004). Development of a multiplex PCR for detection of avian adenovirus, avian reovirus, infectious bursal disease virus, and chicken anemia virus. Mol. Cell. Probes.

[39] Mettifogo E., Buzinhani M., Buim M., Timenetsky J., Ferreira A. (2015). Evaluation of a PCR multiplex for detection and differentiation of *Mycoplasma synoviae*, *M. gallisepticum*, and *M. gallisepticum* strain F-vaccine. Pesqui. Veterinária Bras..

[40] Kaplinski L., Remm M. (2015). MultiPLX: Automatic grouping and evaluation of PCR primers. Methods Mol. Biol..

[41] Ou S.C., Giambrone J.J., Macklin K.S. (2012). Detection of infectious laryngotracheitis virus from darkling beetles and their immature stage (lesser mealworms) by quantitative polymerase chain reaction and virus isolation. J. Appl. Poult. Res..

[42] Creelan J.L., Calvert V.M., Graham D.A., McCullough S.J. (2006). Rapid detection and characterization from field cases of infectious laryngotracheitis virus by real-time polymerase chain reaction and restriction fragment length polymorphism. Avian Pathol..

[43] Williams R.A., Bennett M., Bradbury J.M., Gaskell R.M., Jones R.C., Jordan F.T.W. (1992). Demonstration of sites of latency of infectious laryngotracheitis virus using the polymerase chain reaction. J. Gen. Virol..

[44] Humberd J., García M., Riblet S.M., Resurreccion R.S., Brown T.P. (2002). Detection of Infectious Laryngotracheitis Virus in Formalin-Fixed, Paraffin-Embedded Tissues by Nested Polymerase Chain Reaction. Avian Dis..

[45] Han M.G., Kweon C.H., Mo I.P., Kim S.J. (2002). Pathogenicity and vaccine efficacy of a thymidine kinase gene deleted infectious laryngotracheitis virus expressing the green fluorescent protein gene. Arch. Virol..

[46] van Empel P.C., Hafez H.M. (1999). Ornithobacterium rhinotracheale: A review. Avian Pathol..

[47] Abdelwhab E.M., Lüschow D., Hafez H.M. (2013). Development of Real-Time Polymerase Chain Reaction Assay for Detection of *Ornithobacterium rhinotracheale* in Poultry. Avian Dis..

[48] Hashish A., Sinha A., Sato Y., Macedo N.R., El-Gazzar M. (2022). Development and Validation of a New TaqMan Real-Time PCR for the Detection of *Ornithobacterium rhinotracheale*. Microorganisms.

[49] Veiga I.M.B., Lüschow D., Gutzer S., Hafez H.M., Mühldorfer K. (2019). Phylogenetic relationship of *Ornithobacterium rhinotracheale* isolated from poultry and diverse avian hosts based on 16S rRNA and rpoB gene analyses. BMC Microbiol..

[50] Dieffenbach C.W., Lowe T.M., Dveksler G.S. (1993). General concepts for PCR primer design. PCR Methods Appl..

[51] Werid G.M., Zhang H., Ibrahim Y.M., Pan Y., Zhang L., Xu Y., Zhang W., Wang W., Chen H., Fu L. (2022). Development of a Multiplex RT-PCR Assay for Simultaneous Detection of Four Potential Zoonotic Swine RNA Viruses. Vet. Sci..

[52] Zhao Y., Liu F., Li Q., Wu M., Lei L., Pan Z. (2019). A multiplex RT-PCR assay for rapid and simultaneous detection of four RNA viruses in swine. J. Virol. Methods.

[53] Cao T.B.P., Nguyen V.G., Nguyen T.T., Mai T.N., Vu T.N., Huynh T.M.L. (2022). Application of Direct PCR for Detection of Several Bacteria in Chicken with Respiratory Disease Complex in Hanoi and Neighbour Vicinity. Vietnam J. Agri. Sci..

[54] Nguyen V.G., Chung H.C., Do H.Q., Nguyen T.T., Cao T.B., Truong H.T., Mai T.N., Le T.T., Nguyen T.H., Le T.L. (2021). Serological and Molecular Characterization of Avian Metapneumovirus in Chickens in Northern Vietnam. Vet. Sci..

[55] Van N.T.B., Yen N.T.P., Nhung N.T., Cuong N.V., Kiet B.T., Hoang N.V., Hien V.B., Chansiripornchai N., Choisy M., Ribas A. (2020). Characterization of viral, bacterial, and parasitic causes of disease in small-scale chicken flocks in the Mekong Delta of Vietnam. Poult. Sci..

[56] Gyuranecz M., Foster J.T., Dan A., Ip H.S., Egstad K.F., Parker P.G., Higashiguchi J.M., Skinner M.A., Hofle U., Kreizinger Z. (2013). Worldwide phylogenetic relationship of avian poxviruses. J. Virol..

[57] Clermont O., Christenson J.K., Denamur E., Gordon D.M. (2013). The Clermont *Escherichia coli* phylo-typing method revisited: Improvement of specificity and detection of new phylo-groups. Environ. Microbiol. Rep..

[58] Townsend K.M., Frost A.J., Lee C.W., Papadimitriou J.M., Dawkins H.J. (1998). Development of PCR assays for species- and type-specific identification of *Pasteurella multocida* isolates. J. Clin. Microbiol..

[59] Emam M., Hashem Y.M., El-Hariri M., El-Jakee J. (2020). Detection and antibiotic resistance of *Mycoplasma gallisepticum* and *Mycoplasma synoviae* among chicken flocks in Egypt. Vet. World.

[60] García M., Ikuta N., Levisohn S., Kleven S.H. (2005). Evaluation and comparison of various PCR methods for detection of *Mycoplasma gallisepticum* infection in chickens. Avian Dis..

[61] Chen X., Miflin J.K., Zhang P., Blackall P.J. (1996). Development and application of DNA probes and PCR tests for *Haemophilus paragallinarum*. Avian Dis..

