# Supplementary material for: A Multiplex PCR Method for Simultaneous Detection of Infectious Laryngotracheitis Virus and Ornithobacterium rhinotracheale
Source: Vet Sci. 2023 Apr 3;10(4):272. doi: 10.3390/vetsci10040272 (PMC10144794; doi:10.3390/vetsci10040272)
Supplement: Supplementary file 1 [file vetsci-10-00272-s001.zip › vetsci-2286725-supplementary.pdf]

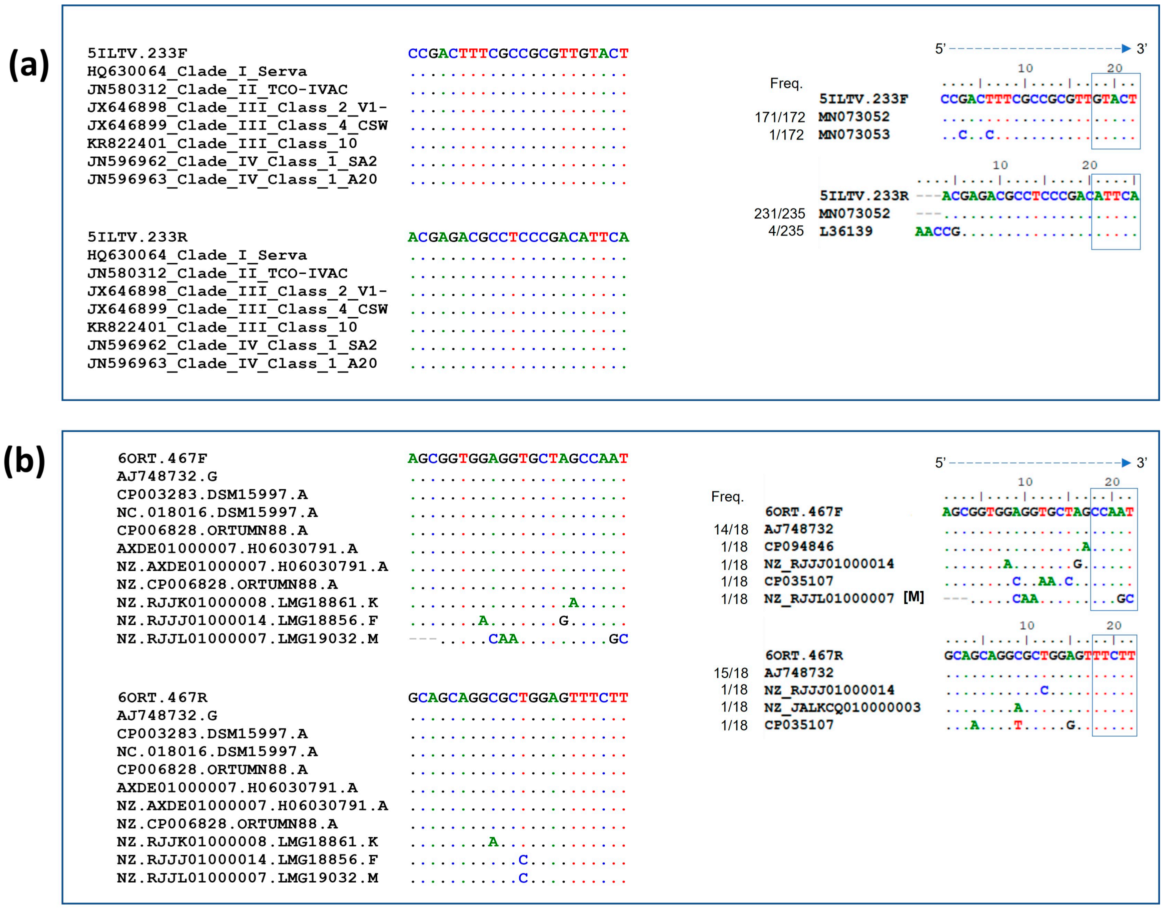

Alignment of binding sites for primers 5ILTV.233F/5ILTV.233R (a) and 6ORT.467F/6ORT.467R (b). Only unique sequences were retained. Boxes indicate the first 5 nucleotides of the 3′ end of each primer. The fractions preceding each primer indicates number of particular sequences out of the homologous sequences available in GenBank. For ILTV primers, the total number of sequences in the alignment with forward (n = 172) and reverse (n = 235) primers differed due to partial sequences included mainly the 3′ fragment. Letters G, A, K, F, M at the end of ORT name indicated serotypes.

Figure S1. alignment of primers.
